# Treatment of Typhoid Fever by Antiseptics, Especially Carbolic and Salicylic Acids

**Published:** 1883-06

**Authors:** 


### Selections.

### Treatment of Typhoid Fever by Antiseptics, Especially Carbolic and Salicylic Acids.

A communication by M. Vulpian to the Academy upon the treatment of typhoid fever by salicylic acid is of the most interesting character. It is an accepted belief that typhoid fever is an infectious disease, like others, with a parasite in the blood which all efforts to overcome have heretofore been vain.

The treatment by quinine, formerly highly recommended by Bean, is now almost forgotten. Sulphate of quinine is, however, one of the most powerful antiseptics. Attention was again called to this fact last year by M. Hallopean. The phenic acid treatment has awakened an enthusiasm which to our mind seems excessive. Indeed, in a recent discussion before the *Societe Medicale des hopitaux*, we observed that now those who had most eagerly accepted the medication by carbolic acid had abandoned it on account of the dangers it involved. On this account M. Siredey, for example, announced that he no longer gave phenic injections except to modify the fetidity of the stools, and immediately after, having given a carbolic acid injection, he gave a large injection of water to cause its evacuation before its absorption. Carbolic acid intoxication is a dangerous phenomenon, and it has always been a matter of astonishment to surgeons to see physicians prescribing internally carbolic acid with an amazing liberality, while they, always on the lookout for absorption by the skin or by the surface of wounds, are constantly busy with the inconveniences of this precious antiseptic.

I may add as a corollary that a number of times I have observed in patients treated medically with these poisons that it has been easy for me to recognize the symptoms at the outset, so familiar have I become by long experience with the action of phenic acid.

All are agreed, however, whether it is true that the acid lowers the temperature or not, that it modifies in no particular the progress of the disease.

M. Vulpian has experimented with other antiseptics, and has employed iodoform, salicylate of bismuth, boric acid and salicylic acid.

Iodoform, boric acid as much as 12 grammes a day, phenate of soda as much per diem as 9 grammes were given without result.

Salicylate of bismuth, 12 grammes a day, lowered the temperature, disinfected the stools, and brought about general improvement, but caused dyspnœa, nasal and intestinal hemorrhages.

M. Vulpian then gave salicylic acid in powder, adopting a very simple form of administration, 25 to 30 centigrammes in wafers every two hours up to 6 or 7 grammes in the twenty-four hours ; each dose should be followed by the ingestion of a liquid, either water or wine. In general the medicine was well borne.

The only accident which seemed to him attributable to the salicylic acid was a little delirium. He met with neither dyspnœa nor hemorrhage. The result was a lowering of temperature as with phenic acid, but more persistent. The general condition was very favorably modified, but the duration of the sickness did not seem to have been materially influenced. M. Vulpian concludes, then, that, without constituting a curative agent of typhoid fever, salicylic acid exercises upon the disease a moderating action of sufficient power to merit a place among the best modes of treatment. He asks the question whether this substance might not be employed as a prophylactic, and whether this substance, incapable of neutralizing the poison when once the system is invaded, will not act to ward it off. It is well known that a healthy man has very well borne 2 grammes of salicylic acid a day.

In the course of the discussion, M. Bordeaudat appropriately called attention to turpentine, a very powerful antiseptic agent, very diffusible and unfortunately neglected in the midst of all these researches.

In conclusion, it is a question which remains to be studied. It is impossible to deny that antiseptics have accomplished something, and agreement upon this conclusion will certainly be reached, if it is not forgotten that antiseptics have very different elective actions and act very differently upon the different micro-organisms. But at any rate, the reader will be struck by this proposition of salicylic acid administered daily as a prophylactic, when it is taken into consideration that it has only been a short time since the employment of this salt was condemned in unmeasured terms. Salycylic acid attacked us on every side, it was said; it is a most dangerous poison, and it endangered, even when administered in the most insignificant doses. The wisest have always thought that there was a singular exaggeration upon this point, and that it was best to limit the employment of an article little enough objectionable, provided it was not ignorantly abused.—*Journal de Medicene et de Chirurgie Practique.*

